# Renin in critically ill patients

**DOI:** 10.1186/s13613-024-01304-3

**Published:** 2024-05-22

**Authors:** Yuki Kotani, Mark Chappell, Giovanni Landoni, Alexander Zarbock, Rinaldo Bellomo, Ashish K. Khanna

**Affiliations:** 1 Department of Intensive Care Medicine, Kameda Medical Center, Kamogawa, Japan; 2 Hypertension and Vascular Research Center, Wake Forest University School of Medicine, Winston-Salem, NC USA; 3 Department of Anesthesia and Intensive Care, IRCCS San Raffaele Scientific Institute, Milan, Italy; 4 School of Medicine, Vita-Salute San Raffaele University, Milan, Italy; 5 Department of Anesthesiology, Intensive Care Medicine and Pain Therapy, University Hospital Muenster, Muenster, Germany; 6 Department of Intensive Care, Austin Hospital, Melbourne, Australia; 7 Australian and New Zealand Intensive Care Research Centre, Monash University, Melbourne, Australia; 8 Department of Critical Care, Melbourne Medical School, The University of Melbourne, Melbourne, Australia; 9 Section On Critical Care Medicine, Department of Anesthesiology, Wake Forest University School of Medicine, Winston-Salem, NC 27157 USA; 10 Perioperative Outcomes and Informatics Collaborative, Wake Forest University School of Medicine, Medical Center Boulevard, Winston-Salem, NC 27157 USA; 11 Outcomes Research Consortium, Cleveland, OH 44195 USA

**Keywords:** Renin, Angiotensin II, Biomarkers, Intensive care units, Mortality, Critical illness, Shock

## Abstract

The renin-angiotensin system (RAS) constitutes one of the principal mechanisms to maintain hemodynamic and fluid homeostasis. However, most research until now on RAS primarily focuses on its relationship with hypertension and its role in critically ill hypotensive populations is not well understood. With the approval of angiotensin II (Ang II) in the United States and Europe, following a phase 3 randomized controlled trial showing efficacy in catecholamine-resistant vasodilatory shock, there is growing interest in RAS in critically ill patients. Among the fundamental components of RAS, renin acts as the initial stimulus for the entire system. In the context of hypotension, its release increases in response to low blood pressure sensed by renal baroreceptors and attenuated negative Ang II feedback loop. Thus, elevated renin could reflect disease severity and predict poor outcomes. Studies investigating this hypothesis have validated the prognostic accuracy of renin in various critically ill populations, with several reports indicating its superiority to lactate for mortality prediction. Accordingly, renin reduction has been used to assess the effectiveness of Ang II administration. Furthermore, renin holds potential to identify patients who might benefit from Ang II treatment, potentially paving the way for personalized vasopressor management. Despite these promising data, most available evidence is derived from retrospective analysis and necessitates prospective confirmation. The absence of a rapid, point-of-care and reliable renin assay presents another hurdle to its integration into routine clinical practice. This narrative review aims to describe the current understanding and future directions of renin as a biomarker during resuscitation of critically ill patients.

## Background

Although hypotension is a major clinical issue in the intensive care unit (ICU), previous research had focused primarily on catecholamines and vasopressin as vasoactive agents. Only recently, however, the results of a phase 3, multinational, randomized controlled trial (RCT) of angiotensin II (Ang II) in catecholamine-resistant vasodilatory shock (ATHOS-3 trial) [1], followed by its approval in the United States and Europe have attracted attention toward RAS from the intensive care community and fostered clinical studies to elucidate its role among critically ill patients. This review aims to summarize recent findings of elevated renin and its downstream derivatives in critically ill patients, with a special focus on its predictive performance of clinically relevant outcomes and its role as a marker of the efficacy of Ang II therapy, and future perspectives on its use in ICU settings.

## Main text

### Pathophysiology of renin-angiotensin system dysfunction

The RAS forms one of the main mechanisms that control blood pressure, fluid balance, and ionic composition (i.e., sodium, chloride, and potassium). Initially, angiotensinogen, synthesized in the liver and released into the circulation, undergoes cleavage by renin—a proteolytic enzyme secreted by renal juxtaglomerular cells—to generate angiotensin I (Ang I) [2]. Due to the very high concentrations of vascular and circulating angiotensin-converting enzyme (ACE), the Ang I decapeptide is immediately processed to Ang II, which is the primary effector peptide of the classical RAS axis [3]. Ang II exerts multiple physiologic actions, including vasoconstriction by binding to the Ang II type I receptor (AT_1_R) [3, 4].

**Fig. 1:**
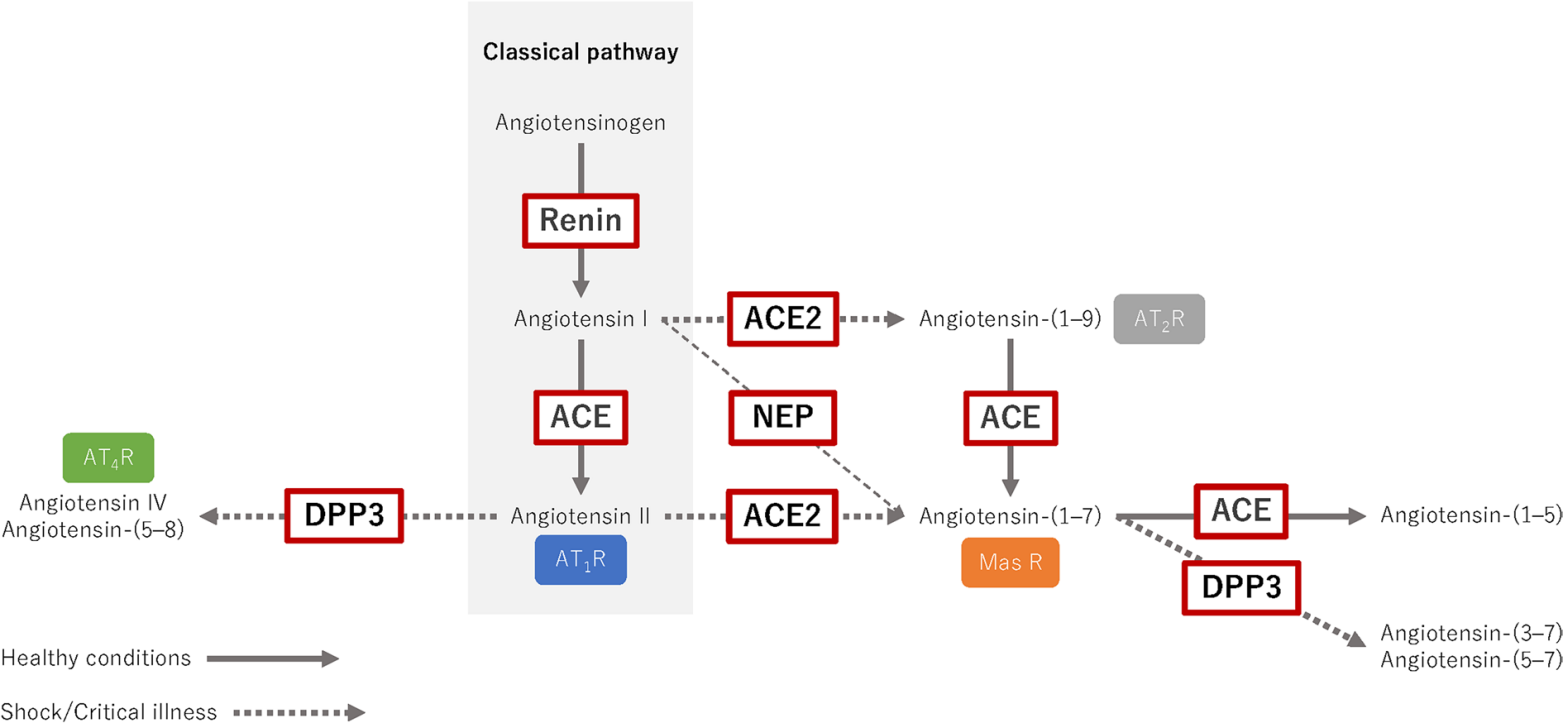
Classical and alternative pathways of the renin-angiotensin system. *ACE* angiotensin-converting enzyme, *AT*_*1*_*R* angiotensin II type I receptor, *AT*_*2*_*R* angiotensin II type II receptor, *AT*_*4*_*R* angiotensin II type IV receptor, *DPP3* dipeptidyl peptidase 3, *NEP* neprilysin

In contrast, there is the potential for an alternative pathway that counterbalances the classical axis, primarily through the effector molecule angiotensin-(1–7) (Ang-(1–7)). The synthesis of Ang-(1–7) includes its transformation from Ang II by ACE2 [5] and direct generation from Ang I by the action of metalloendopeptidases such as neprilysin [6, 7] (Fig. 1). By binding to its unique Mas receptor, Ang-(1–7) can mitigate the physiological and pathological effects of the classic pathway, thereby exhibiting vasodilatory, natriuretic, anti-inflammatory, and anti-fibrotic effects [8]. Conditions that influence the binding of Ang II to the AT_1_R versus ACE2-dependent metabolism of the peptide involve the relative concentrations of the AT_1_R and ACE2 in a particular tissue under normal or pathological situations [9]. The AT_1_R exhibits a greater preference for Ang II (K_D_ of ~ 1 nM) than ACE2 (K_M_ of ~ 2 µM) [9]. However, pathological conditions such as septic shock may reduce AT_1_R expression and increase ACE2 that would favor processing of Ang II to Ang-(1–7). Reduced levels of ACE in septic shock may also promote the direct conversion of Ang I to Ang-(1–7) by neprilysin [9]. ACE2 also cleaves Ang I to Ang-(1–9) that may stimulate the Ang II type II receptor, which shares similar actions of Ang-(1–7) to antagonize the classical Ang II-AT_1_R pathway [10]. In contrast to ACE2, ACE metabolizes Ang-(1–7) to Ang-(1–5) and is the primary route for degradation of the peptide in the circulation [9].

Dipeptidyl peptidase 3 (DPP3), a cytosolic zinc-dependent amino dipeptidase ubiquitously expressed in human cells and tissues, plays a role in modulating peptides involved in both the classical and alternative RAS pathways. Under normal conditions, intracellular levels of DPP3 act as a regulator of the oxidative stress and immunological response [11]. However, in critical illness, the cellular release of DPP3 due to tissue injury increases circulating levels of the peptidase that may contribute to the metabolism of vasoactive peptides [11]. Although we find that human proximal tubule cells released DPP3 into the cell media and subsequently utilized these cells to purify and characterize human DPP3, our studies did not identify processes involved in the secretion of the aminopeptidase [12, 13]. Indeed, to our knowledge, the factors that govern the expression and regulated release of the enzyme are currently unknown [11, 13]. DPP3 catalyzes the hydrolysis of Ang-(1–7) into Ang-(3–7), which is immediately converted to Ang-(5–7), while DPP3 cleaves Ang II to Ang IV (Ang-(3–8)), and then Ang-(5–8) (Fig. 1) [13]. Ang IV binds to Ang II type IV receptor to exert vasodilation, cardioprotective effects, and natriuresis [14]. DPP3 does not cleave larger peptides (≥ 10 amino acids) such as Ang I [9, 13, 15, 16].

Under physiological conditions, triggers such as hypotension, sympathetic nervous system stimulation, and reduced Ang II generation (loss of negative feedback), may provoke renin secretion. Conversely, critically ill hypotensive patients often exhibit impaired RAS signaling characterized by downregulation of AT_1_R expression [17] and a reduced ACE activity [18]. These alterations may further worsen hypotension and dysregulated immune effects via lack of endogenous Ang II or its active site of action.

### Outcome prediction

RAS dysregulation during critical illness has been documented for several decades [19]. A broad review of observational data has suggested that the predictive performance of serum renin is reliably high across various critically ill and perioperative settings [20]. Since decreased blood pressure and reduced organ perfusion are major triggers for renin secretion, hypotensive patients are the most studied population, and most data showed an association of serum renin with mortality [21–26]. Of these studies, three identified renin as an independent mortality risk factor after multivariable adjustment [21, 22, 25]. For example, a monocentric prospective study of 20 heterogenous shock patients reported a significantly greater change of serum renin levels in non-survivors than survivors (92 ± 57 vs. −32 ± 57 µU/mL) [23]. Another study of 53 hypotensive patients on vasopressors found that the initial renin value after ICU admission and renin changes could predict hospital mortality [25]. A recent study also assessed the relationship between renin and mortality using the data of a previous RCT on a cocktail therapy of vitamins and corticosteroids in sepsis (VICTAS trial) [27]. This study found not only initial serum renin values but a relative increase in renin from day 0 to day 3 were associated with 30-day mortality after adjusting relevant confounding factors. Interestingly, patients had a survival advantage when their renin decreased over time compared to those whose values of this biomarker increased. This first of kind analysis also examined other components of the RAS pathway and did not find an association of Ang II, ACE 2 and Ang (1–7) with mortality. While lactate has been suggested for use to guide resuscitation in the surviving sepsis guidelines [28] and is widely used in ICUs, especially among high-income countries, using lactate as a resuscitation target requires cautious evaluation since hyperlactemia may occur for reasons other than tissue hypoperfusion or increased production (e.g., hypoperfusion and excessive β adrenergic stimulation via epinephrine use) or suppressed excretion due to liver failure [29]. Notably, two studies compared the predictive performance for mortality between renin and lactate [23, 25]. The area under the receiver operating characteristics (AUROC) was 0.80 for maximum renin level and 0.70 for maximum lactate level for the first study [23], while that for initial renin and lactate was 0.682 and 0.615, respectively in the latter study [25]. Therefore, Jeyaraju and colleagues demonstrated that both absolute renin and lactate predict mortality whereas only an increase of renin and not lactate over 72 h had an independent association with mortality [25]. In addition, there was a higher proportion of renin > 40 pg/mL than lactate > 2 mmol/L in non-survivors than survivors [25].

From a physiological point of view, circulating renin quickly and directly reflects hemodynamic changes because hypotension is the main trigger of its secretion, and the half-life of renin is short (approximately 10 min). Taken together, these preliminary data may suggest the potential utility of renin as a promising perfusion marker.

Apart from mortality, renin can also predict adverse renal outcomes [24, 30–32]. In a small study of 41 septic shock patients, plasma renin concentration was associated with receipt of renal replacement [24]. A prospective cohort study of 197 cardiac surgery patients investigated if changes in circulating renin levels between before and 4 h after surgery could predict postoperative AKI within 72 h [31]. When divided into two groups with the median renin increase (3.7 µU/mL) as reference, patients with a greater renin increase after surgery were more likely to experience postoperative AKI. The predictive value of postoperative renin was relatively high (AUROC of 0.80). The relationship between renin and major adverse kidney events (MAKE) at hospital discharge was assessed in 280 heterogenous ICU patients [30]. MAKE was defined as a composite endpoint of mortality, renal replacement therapy dependence, and reduction in estimated glomerular filtration rate to ≤ 75%. When 280 patients were categorized into three groups according to serum renin levels (median renin levels of 7.2 pg/mL in the first tertile, 40.7 pg/mL in the second tertile, and 355.3 pg/mL in the third tertile), multivariable logistic regression analyses showed that compared to the lowest renin group, the intermediate and high renin groups were at a higher risk of MAKE at hospital discharge.

When assessing renin in vasodilatory shock, cautious consideration should be given to previous use of RAS inhibitors (i.e., ACE inhibitors and Ang II receptor blockers [ARBs]). A single-center prospective cohort study found that recent exposure to RAS inhibitors was significantly associated with an increased renin levels, nearly doubling them [33]. A post-hoc analysis of a phase 3 RCT on Ang II in vasodilatory shock was also conducted to evaluate the relationship between previous RAS inhibitors use and renin levels [34]. When compared to patients without recent RAS inhibitor exposure, those who had received ACE inhibitors exhibited higher renin levels at baseline [34]. Furthermore, exogenous Ang II administration reduced renin levels at 3 h in patients without RAS inhibitor exposure and those with ACE inhibitor exposure but not in those with ARBs exposure [34]. These observations align with physiological negative feedback of RAS and pharmacological mechanisms of action of ACE inhibitors, ARBs, and Ang II.

Current evidence suggests that renin release from juxtaglomerular cells of the kidney is triggered by hypoperfusion of the renal medulla or decreased endogenous Ang II or a combination of these factors. In addition, a baseline, a threshold difference and a change in renin may outperform lactate in terms of mortality prediction. However, no dedicated data is available as to whether renin can reflect the balance between tissue oxygen demand and supply.

### Treatment response

Given that renin release is regulated by a negative feedback loop by Ang II generation, it is a reasonable hypothesis that increased serum renin concentrations could serve as an indicator of reduced Ang II levels and or a dysfunctional and unstable RAS, thereby acting as a potential trigger for exogenous Ang II administration. Furthermore, decreased serum renin levels after Ang II infusion may imply a degree of efficacy of Ang II therapy because of an association of renin with adverse clinical outcomes [20].

#### Vasodilatory shock

The ATHOS-3 trial which randomly assigned catecholamine-resistant vasodilatory shock patients either to Ang II or to placebo (which included continuation of background non-Ang II vasopressors). Patients who received Ang II had a sharp and significant decline in serum renin in the first 3 h compared to the placebo group where renin was unchanged. A secondary analysis was performed to investigate the relationship between Ang II treatment and renin changes between randomization and 3 h after the study drug initiation [35]. Serum renin levels at randomization were similar in the two groups (146.1 pg/mL in the Ang II group and 193.7 pg/mL in the placebo group, P = 0.42). At 3 h, greater renin reduction was observed in the Ang II group (median, 54.3%; interquartile range [IQR], 37.9 to 66.5% reduction) than in the placebo group (median, 14.1%; IQR, 37.6% reduction to 5.1% increase), which was statistically significantly different (P < 0.0001). Furthermore, to analyze the effect of Ang II on mortality in relation to baseline renin levels, patients were divided into high and low renin groups using the median value as the threshold (172.7 pg/mL). The previously mentioned VICTAS post hoc analysis also showed a very similar median value of 188.7 pg/ml that dichotomized to high renin that was associated with mortality versus not [27]. Of note, compared to placebo, Ang II administration was significantly associated with reduced 28-day mortality in patients with high baseline renin values (hazard ratio [HR], 0.56; 95% confidence interval [CI] 0.35–0.88; P = 0.01) but not in those with low baseline renin values (HR, 1.11; 95% CI 0.66–1.86; P = 0.70). Multivariable analyses confirmed that increased renin at randomization was an independent risk factor for 28-day mortality and that Ang II administration was associated with mortality risk reduction in patients with high baseline renin values.

Elevated renin levels, rather than high vasopressor doses, appear to indicate the population who could likely benefit from Ang II therapy. Since high renin levels suggest inadequate circulating Ang II [35], renal hypoperfusion, or an inadequate conversion of renin to Ang II [27], exogenous Ang II administration could restore the dysfunctional RAS balance and reduce mortality in high-renin patients. Notably, in ATHOS-3, all the randomized patients received at least 0.2 µg/kg/min of norepinephrine equivalent vasopressors, but the lowest quartile renin value was 58 pg/mL in these patients [35]. In other words, only 25% of the study participants exhibited renin below that threshold [35]. Similarly, in VICTAS, the lowest quartile was 27 pg/mL [27]. Furthermore, in another cohort study on Ang II in vasodilatory shock, 4 out of 40 patients had renin levels < 40 pg/mL [33]. Given that normal renin level is typically considered < 40 pg/mL, the proportion of patients with low renin levels in septic shock or vasodilatory shock appears to range from 10 to 25% and this group may not be responsive to exogenous Ang II.

Regarding the relationship between baseline vasopressor requirements and Ang II therapy, another post-hoc analysis of ATHOS-3 showed that a subgroup suffering from more severe shock (i.e., norepinephrine equivalence > 0.25 µg/kg/min), did not benefit from Ang II treatment; while the other subgroup (norepinephrine equivalence was between 0.2 and 0.25 µg/kg/min) experienced reduced mortality when assigned to Ang II [36]. Thus, Ang II therapy may carry survival benefits in patients who show high renin values or have not reached high norepinephrine equivalence.

Despite the retrospective design, these observations suggest valuable insights into RAS dynamics and the potential utility of renin-guided Ang II therapy in catecholamine-resistant vasodilatory shock patients.

#### Cardiac surgery

Several studies have evaluated the effect of Ang II treatment on renin concentrations among cardiac surgery populations. The first was a retrospective cohort study in 40 cardiac surgery patients who had an increase in renin at 4 h after cardiopulmonary bypass compared to preoperative levels (> 3.7 µU/mL) and received continuous norepinephrine infusion [32]. The cutoff for the renin increase was defined by evidence from previous literature that this value defined a higher risk of worse outcomes [31]. Among these patients, 20 received Ang II in addition to norepinephrine in the postoperative course and 20 underwent usual care without Ang II. When adjusted by the renin level at 4 h after surgery, renin levels at 12 h after surgery were significantly lower in the Ang II group than in the usual care group (71.7 µU/mL [IQR, 21.9–211.4] vs.130.6 µU/mL [IQR, 62.9–317.0]; adjusted P = 0.034). In addition, Ang II administration, compared with usual care, was associated with reduced cumulative norepinephrine dose (1.33 mg [IQR, 0.78–2.04] vs. 3.25 mg [IQR, 1.00–4.75]; P = 0.046).

The first randomized trial comparing the intraoperative infusion of Ang II with that of norepinephrine in cardiac surgery patients was performed as a feasibility trial [37]. Following the confirmatory results regarding adherence without any concerns for adverse events [37], the authors explored the effect of Ang II on RAS within this randomized trial [38]. Compared to baseline, the renin level at ICU admission significantly increased in the norepinephrine group (52 [IQR, 115–137] vs. 112 [IQR, 49–410] µU/mL; P < 0.001), but not in the Ang II group (87 [IQR, 40–232] vs. 82 [IQR, 24–172] µU/mL; P = 0.36). At 24 h following surgery, renin levels increased in both groups. An important limitation was that only 4/28 patients in the Ang II group received the study drug longer than 24 h. An additional analysis was also performed to test whether the preoperative RAS inhibitor use affected the renin kinetics and it found significantly higher renin levels before surgery in patients with preoperative RAS inhibitor therapy than those without.

Considering the physiological background and preliminary data suggesting that renin could identify patients who would benefit from Ang II therapy, a randomized trial tested the hypothesis that Ang II reduces the kidney stress in vasoplegic cardiac surgery patients with a renin increase after surgery [39]. Among 63 patients, 31 were randomized to the Ang II group and 32 to the placebo group. Although kidney stress, measured by the difference in Nephrocheck® values (tissue inhibitor of metalloproteinases-2 [TIMP-2])*(insulin-like growth factor-binding protein 7 [IGFBP7]), was not significantly different (median, 0.06 vs. -0.08 [ng/mL]^2^; P = 0.19), Ang II, compared to placebo, reduced fluid administration (2946 vs. 3341 mL; P = 0.03) and norepinephrine dose (0.19 vs. 4.18 mg; P < 0.001) [39].

Table 1 summarizes the findings regarding renin in the context of randomized trials in intensive care settings.

**Table 1:** Data on renin in randomized trials of critically ill settings

First author, year	Population	Patients	Intervention	Comparator	Results
Bellomo R 2020 [35]	Refractory vasodilatory shock	255	Angiotensin II	Placebo	Compared to placebo, Ang II therapy was associated with greater reduction in renin levels In the high renin group (> 172.7 pg/mL), Ang II therapy was associated with reduced mortality
Coulson T 2023 [38]	Cardiac surgery	60	Angiotensin II	Norepinephrine	Renin release was suppressed in the Ang II group compared to the norepinephrine group
Busse LW 2023 [27]	Sepsis and septic shock	103	Vitamin C, thiamine, hydrocortisone	Placebo	Baseline renin values and a relative renin change over three days were associated with mortality
Sadjadi M 2024 [39]	Cardiac surgery	60	Angiotensin II	Placebo	Renin increase between preoperative and postoperative values (> 3.7 µU/mL) was used as an inclusion criterion No significant difference was observed in kidney stress detected by renal biomarkers

### Logistic and economic issues

The biggest challenge to incorporate renin into ICU-based treatment protocols is a limited availability of a point-of-care renin assay that completely distinguishes prorenin [40]. Given the urgency of shock management, the results of renin tests should also be available at least within one hour, ideally within 10 min as typically performed with lactate. This is not yet possible although promising technology using enzyme-linked immunosorbent assays (ELISA) based techniques may be on the horizon.

Another consideration is the method of measurement of renin levels. A scoping review of renin in critically ill and perioperative settings identified heterogeneity in the measurement methods (i.e., renin activity vs. renin concentration) [20]. Moreover, plasma renin activity values are influenced by angiotensinogen, prorenin, and sample handling (i.e., cryoactivation of prorenin) and the required steps to generate and quantify Ang I levels [9]. On the other hand, newly developed dual antibody-based ELISAs, are a preferable method to directly determine active circulating renin concentrations, which can differentiate renin and prorenin and does not require an Ang I generation step from either endogenous (plasma renin activity) or exogenous angiotensinogen (plasma renin concentration) that greatly simplifies the assay [9].

Conducting a cost–benefit analysis is crucial, particularly in the context of incorporating novel technologies into standard clinical practice. The measurement of serum or plasma renin levels, which can be conducted in most laboratories at a relatively low cost (around 10 euros per assay), offers a notable example. Given the documented potential of renin to predict adverse outcomes [23, 25, 31] and identify patients who could respond favorably to Ang II treatment [35], the routine assessment of serum renin levels may enhance patient care quality. However, no economic evaluation has yet been conducted regarding the use of renin measurement in intensive care settings.

### Future perspective

Based on the current evidence, potential clinical application of renin in critically ill patients include prognostication, guide for Ang II initiation, and real time dynamic resuscitation targets. Moreover, renin can be used in clinical research as an inclusion criterion and surrogate endpoint (Table 2).

**Table 2:** Potential application of renin in critically ill settings

Role	Explanation
Prognostic factor	Renin is associated with mortality, adverse renal outcomes, and hemodynamic instability Renin may predict mortality better than lactate
Guide to start angiotensin II	High renin levels may indicate the effectiveness of angiotensin II administration
Resuscitation target	Compared to lactate, renin may predict mortality better and be less influenced by reasons other than hypoperfusion
Inclusion criteria	Using renin as an inclusion criterion will identify patients at risk of developing such complications
Outcome measures	The association of renin with mortality suggests its utility as a surrogate outcome

To further clarify the role of renin in critically ill patients, there are several research topics to be addressed. First, there are still uncertainties regarding renin kinetics. Given the rapid changes in hemodynamic conditions and therapeutic interventions during the initial phase of shock, serial renin measurements within that period may help guide management. However, previous studies have measured renin levels once or twice during the initial 24 h [20]. A study with two measurements within the 24-h window reported renin at randomization and 3 h after the study drug initiation, which highlights the need for more frequent measurements to elucidate how renin changes over time [35]. Moreover, whether absolute or relative renin values are better is also a matter of investigation. In cardiac surgery patients, relative changes in renin before and after surgery were better than the postoperative absolute value in terms of AKI prediction [31]. However, no study has specifically compared the predictive validity between absolute values and relative changes within a short duration in other populations.

Second, renin may help guide hemodynamic management. Since renin may outperform lactate in predicting mortality [23, 25], using renin instead of lactate as a resuscitation target could improve clinical outcomes. Future research should address the optimal reliable renin cutoff value for successful resuscitation. In addition, bedside renin assay availability is essential to allow for serial measurements and timely treatment adjustment according to renin levels.

Third, high renin levels could identify patients who may benefit from Ang II administration given the data from ATHOS-3 trial [35]. Although a randomized trial did not show renoprotective effects of Ang II as the first-line vasopressor in hyperreninemic cardiac surgery patients, any definitive conclusion cannot be made due to its small sample size [39]. Finally, we need to evaluate and establish the most complete assessment of renin in the plasma of septic shock patients that associates with disease severity and outcome by using a combination of assays (prorenin, renin, ACE, aldosterone and their ratios) to plasma renin activity.

## Conclusions

Recent studies have shed a light on the role of RAS in the management of critically ill patients. Renin is consistently associated with poor outcomes across different patient categories and its predictive accuracy may outperform that of lactate. Renin may help identify patients who are likely to benefit from Ang II infusion, which provides a promising hypothesis for future randomized trials. The development of point-of-care renin assay is crucial for its application in clinical settings.

## Data Availability

The data presented in this manuscript are available from the corresponding author upon reasonable request.

## Abbreviations

ACE: Angiotensin-converting enzyme
AKI: Acute kidney injury
Ang I: Angiotensin I
Ang II: Angiotensin II
AT_1_R: Angiotensin II type I receptor
AUROC: Area under the receiver operating characteristics
DPP3: Dipeptidyl peptidase 3
ELISA: Enzyme-linked immunosorbent assay
HR: Hazard ratio
ICU: Intensive care unit
IGFBP7: Insulin-like growth factor-binding protein 7
IQR: Interquartile range
MAKE: Major adverse kidney events
RAS: Renin-angiotensin system
RCT: Randomized controlled trial
TIMP-2: Tissue inhibitor of metalloproteinases-2
